# The adaptation problems of patients undergoing hemodialysis: socio-economic and clinical aspects^1^

**DOI:** 10.1590/0104-1169.3525.2504

**Published:** 2014

**Authors:** Cecília Maria Farias de Queiroz Frazão, Jéssica Dantas de Sá, Ana Beatriz de Almeida Medeiros, Maria Isabel da Conceição Dias Fernandes, Ana Luisa Brandão de Carvalho Lira, Marcos Venícios de Oliveira Lopes

**Affiliations:** 2 Doctoral student, Universidade Federal do Rio Grande do Norte, Natal, RN, Brazil; 3 Undergraduate student in Nursing, Universidade Federal do Rio Grande do Norte, RN, Brazil; 4 Master's student, Universidade Federal do Rio Grande do Norte, Natal, RN, Brazil. Professor, Departamento de Enfermagem, Universidade Federal do Rio Grande do Norte, Natal, RN, Brazil; 5 PhD, Adjunct Professor, Departamento de Enfermagem, Universidade Federal do Rio Grande do Norte, Natal, RN, Brazil; 6 PhD, Associate Professor, Departamento de Enfermagem, Universidade Federal de Ceará, Fortaleza, CE, Brazil

**Keywords:** Nursing, Nursing Theory, Renal Dialysis

## Abstract

**OBJECTIVES:**

to identify adaptation problems under Roy's Model in patients undergoing hemodialysis and to correlate them with the socioeconomic and clinical aspects.

**METHOD:**

a transversal study, undertaken using a questionnaire. The sample was made up of 178 individuals. The Chi-squared and Mann-Whitney U tests were undertaken.

**RESULTS:**

the adaptation problems and the socioeconomic and clinical aspects which presented statistical associations were: Hyperkalemia and age; Edema and income; Impairment of a primary sense: touch and income; Role failure and age; Sexual dysfunction and marital status and sex; Impairment of a primary sense: vision and years of education; Intolerance to activity and years of education; Chronic pain and sex and years of education; Impaired skin integrity and age: Hypocalcemia and access; Potential for injury and age and years of education; Nutrition below the organism's requirements and age; Impairment of a primary sense: hearing and sex and kinetic evaluation of urea; Mobility in gait and/or coordination restricted, and months of hemodialysis; and, Loss of ability for self-care, and months of hemodialysis and months of illness.

**CONCLUSION:**

adaptation problems in the clientele undergoing hemodialysis can be influenced by socioeconomic/clinical data. These findings contribute to the development of the profession, fostering the nurse's reflection regarding the care.

## Introduction

As Nursing has advanced as a profession, the need has arisen for a conceptual landmark upon which the work's organization can be based, with the objective of understanding issues related to the client, the environment, the goal, and to qualified care^(^ 1 ^)^.

In the ambit of the profession's conceptual landmark, there is the adaptation model proposed by Callista Roy. In this model, the nurse undertakes the role of mediator for the promotion of the client's positive adaptation, developing the nursing process in six phases, namely: assessment of the behavior, assessment of stimuli, nursing diagnosis, and establishment of goals, intervention and assessment^(^ 2 ^)^. This process contributes to nursing care which is systematized and geared towards the patient's adaptation to the limitations imposed by the clinical situation. 

Among the varying clinical situations, emphasis is placed on chronic kidney disease (CKD), as patients with this condition are exposed to varying adaptation problems resulting from alterations in their routine. The main alterations are: restrictions on water and food ingestion, a continuous program of medication and dependence on the hemodialysis machine for maintenance of life^(^ 3 ^-^ 5 ^)^. Corroborating this statement, one study with this clientele confirmed that there is a relationship between the development of chronic kidney disease and social and environmental alterations^(^ 6 ^)^.

In this context, the nurse, through Roy's nursing process, contributes to the promotion of the client's positive adaptation. Thus, emphasis is placed on the importance of using this model in clinical practice, principally in the area of nephrology, bearing in mind the alterations in the physiological, psychological, spiritual and social contexts, caused by the CKD and its treatment. 

In this perspective, discovering ways of caring for these people, guided by Roy's theoretical model, will help nurses in the identification of the most frequent adaptation problems in this clientele. Furthermore, to understand them in conjunction with the socioeconomic and clinical situation in which they are inserted entails a greater role for the nurse, who, based on this analysis, can take the social reality of the subject into account in the planning of the care, for the transformation of the problems into positive responses.

Therefore, perceiving the consideration of the being who receives the nursing care as a person in constant interaction with their environment as an advantage in the adaptation model, focusing on the need for adjustment to these changes, and recognizing the importance of the nursing process, the question is raised: What are the adaptation problems of the type addressed by Roy present in chronic kidney patients receiving hemodialysis? Are these problems correlated with the socioeconomic and clinical aspects? Thus, this study's object is to identify the adaptation problems of the type addressed by Roy in patients receiving hemodialysis, and to correlate them with the socioeconomic and clinical aspects. 

## Method

This is a transversal study, undertaken in a clinic which is a center of excellence in dialysis, in a city in the Northeast of Brazil. The population was made up of 330 patients who are registered and receiving hemodialysis in the above-mentioned clinic. For the sample, the formula for finite populations was applied, taking into account the study's level of confidence of 95% (Zα =1.96), sample error of 5%, population size of 330 persons and the prevalence of nursing diagnoses with a conservative value corresponding to 50%. Based on the application of the formula, a sample size of 178 individuals was found. 

Patient selection was through a consecutive convenience sample. The following inclusion criteria were adopted: to have a medical diagnosis of chronic kidney disease; to be registered and receiving hemodialysis in the above-mentioned clinic; to be aged between 20 and 65 years old; and to have been advised and have conditions to participate. The exclusion criteria were: chronic kidney patients with other diseases unrelated to the renal situation which could alter the profile of the adaptation problems, such as: cancer, neurological disease, advanced heart disease, advanced lung disease, progressive liver disease, cerebrovascular disease, coronary disease and extensive peripheral disease. This last criteria was adopted, as behaviors, stimuli and adaptation problems are influenced by the physiopathology of each disease^(^ 2 ^)^. Thus, it is believed that the patient with chronic kidney disease alone has adaptation problems which are specific to this condition. 

For data collection, an instrument was constructed of the questionnaire type, based in the three first phases of the nursing process (assessment of behavior, assessment of stimuli, nursing diagnosis) proposed in Roy's theoretical model, in the consultations of the literature related to the techniques of clinical evaluation, and in the publications on chronic kidney disease and nursing diagnoses^(^ 2 ^,^ 7 ^-^ 8 ^)^. The above-mentioned instrument was made up of sections, namely: sociodemographic data; history of the current health problems; the hemodialysis treatment; the adaptation modes of Roy (physiological, self-concept, role function and interdependence); the general physical examination, and by body areas; and the values of the urea, creatinine, calcium, potassium, hemoglobin, hematocrit, parathormone, urea reduction ratio (KT/V) and phosphorus. It is emphasized that these last data were collected in the laboratory tests present in the patients' medical records.

In order to undertake content and face validation, the instrument was presented to two lecturers who undertake studies on the systematization of nursing care. The lecturers' suggestions were incorporated into the instrument, which was later applied in the form of a pre-test to 18 chronic kidney patients receiving hemodialysis. There was no need for the instruments to be altered. As a result, those who participated in the pre-test were included in the sample of this study. 

Data collection occurred during the hemodialysis session, in October 2011 - February 2012, undertaken by three nurses and five students receiving scientific initiation grants, of the last year of the undergraduate Nursing course, who had been previously trained. The training course lasted 10 hours, during which there was a review regarding the physiopathology of chronic kidney disease, the alterations experienced by the patient receiving hemodialysis, Roy's theoretical model and the general physical examination, and examination by body area. The course was administered by three nurses and coordinated by the professor supervising the project. So as to ensure the patient's privacy, questions regarding self-concept, role function and interdependence were put in a low voice. 

In organizing the data, an individual process of clinical judgment of the adaptation problems evidenced by the patients was undertaken, in two phases: the analysis, which involved the categorization of the data and the identification of gaps; and the summary, formed by the grouping, comparison, identification and relating of the etiological factors^(^ 9 ^)^. It is emphasized that, where gaps were present in the first phase, the patient was approached a second time. After this stage, the results obtained underwent a peer review process among the authors, so as to ensure a consensus judgment, the aim being, in this way, to obtain greater accuracy in the inference of the adaptation problems. 

Following that, a database was constructed, in which the socioeconomic, clinical, and adaptation problems identified were recorded. For the data analysis, a statistical program was used: IBM's Statistical Package for the Social Sciences (SPSS), version 16.0, creating descriptive data of means and standard deviation for the quantitative variables, and the p-value for the Chi-squared and Mann-Whitney U tests, with the aim of ascertaining the existence of statistical associations. Thus, for the statistical significance of the tests specified, a level of 5% (p< 0.05) was adopted.

This study was approved by the Research Ethics Committee of the institution responsible for the research (Protocol N. 115/11) and received the Certificate of Presentation for Ethical Appreciation (N. 0139.0.051.000-111). The patients showed their acceptance to participate in the study through signing the terms of consent. 

## Results

Among the patients interviewed, 52.2% were male, 62.9% had a partner, and the mean age was 46.6 years old (±12.3). Regarding family income, 92.1% received one minimum salary, with variation from one to 30 salaries (the value of R$622.00 was considered as the minimum Brazilian salary at the time of the study). In relation to education, the mean was 8.5 years of study (±4.8).

In relation to the clinical data, the predominant vascular access was the arteriovenous fistula (93.8%), and the time since diagnosis of CKD and the time of undertaking hemodialysis therapy presented medians of six and four years, respectively. Regarding the value of the urea reduction ratio (KT/V), a mean of 1.5 (±0.6) was obtained.

For the adaptation problems addressed by Roy, the mean per patient was of 6.4 (±2.3). The total was 22, namely: Retention of intracellular liquid (99.4%); Hyperkalemia (64.6%); Hypothermia (61.8%); Edema (53.9%); Intolerance to activity (47.2%); Role failure (42.7%); Potential for injury (37.1%); Mobility - gait and/or coordination restricted (35.4%); Hypocalcemia (34.8%); Sexual dysfunction (28.7%); Impairment of the primary sense: vision (28.1%); Sleep deprivation (25.3%); Chronic Pain (15.7%); Impairment of primary sense: hearing (15.2%); Low self-esteem (12.4%); Acute Pain (11.2%); Loss of ability for self-care (11.2%); Impaired skin integrity (6.7%); Constipation (5.6%); Impairment of a primary sense: touch (2.8%); Nutrition below the organism's requirements (1.1%); and Diarrhea (1.1%). 

The results regarding the analysis of association between the clinical aspects and the adaptation problems addressed by Roy are presented in Table 1. 

**Table 1 -:** Analysis of the association between socioeconomic and clinical aspects and the adaptation problems addressed by Roy in patients with CKD receiving hemodialysis (HD). Natal, RN, Brazil, 2013

Adaptation problems	Age*	Years of study*	Income*	Sex^†^	Marital status^†^	Access^†^	Months with CKD*	Months in HD*	KT/V*
Hyperkalemia	0.020^‡^	0.383	0.057	0.548	0.237	0.679	0.784	0.481	0.152
Edema	0.453	0.824	0.016^‡^	0.145	0.454	0.101	0.650	0.104	0.541
Impairment of a primary sense: touch	0.072	0.156	0.006^‡^	0.208	0.423	0.953	0.500	0.090	0.484
Role failure	0.033^‡^	0.628	0.423	0.318	0.568	0.672	0.876	0.086	0.955
Sexual dysfunction	0.827	0.338	0.829	0.009^‡^	0.003^‡^	0.293	0.176	0.853	0.152
Impairment of a primary sense: sight	0.879	0.000^‡^	0.060	0.531	0.852	0.411	0.089	0.857	0.504
Intolerance of activity	0.093	0.037^‡^	0.599	0.790	0.564	0.372	0.847	0.895	0.095
Chronic Pain	0.162	0.045^‡^	0.075	0.007^‡^	0.108	0.657	0.082	0.089	0.217
Impaired skin integrity	0.024^‡^	0.170	0.249	0.662	0.781	0.350	0.814	0.330	0.882
Potential for injury	0.003^‡^	0.032^‡^	0.314	0.870	0.227	0.160	0.963	0.179	0.195
Nutrition below the organism’s requirements	0.028^‡^	0.753	0.117	0.174	0.064	0.988	0.836	0.464	0.229
Hypocalcemia	0.762	0.241	0.470	0.613	0.747	0.030^‡^	0.806	0.990	0.214
Impairment of a primary sense: hearing	0.926	0.977	0.397	0.041^‡^	0.390	0.553	0.597	0.869	0.032^‡^
Retention of intracellular liquid	0.365	0.479	0.927	0.294	0.441	0.996	0.182	0.325	0.775
Hypothermia	0.941	0.178	0.946	0.649	0.126	0.242	0.752	0.300	0.975
Sleep deprivation	0.919	0.086	0.863	0.607	0.639	0.590	0.863	0.107	0.830
Low self-esteem	0.068	0.141	0.837	0.649	0.108	0.628	0.069	0.351	0.419
Acute Pain	0.866	0.450	0.105	0.491	0.204	0.648	0.133	0.176	0.090
Mobility – gait and/or coordination restricted	0.068	0.216	0.796	0.219	0.132	0.265	0.419	0.008^‡^	0.721
Loss of ability for self-care	0.146	0.993	0.275	0.831	0.774	0.260	0.016^‡^	0.001^‡^	0.718
Constipation	0.246	0.079	0.947	0.425	0.844	0.874	0.319	0.133	0.804
Diarrhea	0.863	0.845	0.994	0.949	0.064	0.988	0.557	0.857	0.229

* Mann-Whitney U Test.

† Chi-squared Test.

‡ Variables which presented statistical association.

## Discussion

Based on the data obtained following the statistical analysis between the adaptation problems and the socioeconomic and clinical variables of the clientele investigated, some associations were observed. Among these, the Roy adaptation problem of hyperkalemia had a statistical association with the variable of age. 

Hyperkalemia is characterized by a rise in the serum concentration of potassium. However, differing from the findings of this research, one study undertaken with chronic kidney patients showed a relationship of hyperkalemia with the decline of the renal function present in the condition, rather than with the advance of age^(^ 10 ^)^.

The adaptation problem of edema had statistical association with the variable of income. Edema and weight gain in a short period are considered to be signs commonly identified in the population with kidney disease, as they are a consequence of the inefficient maintenance of the sodium balance by the organism^(^ 11 ^)^. It stands out, furthermore, that the association of the edema with cardiovascular risk factors is an important predictor for mortality in these patients, it being relevant to observe this sign^(^ 12 ^)^.

Income was also associated with the impairment of a primary sense: touch. Alterations in the basal and intermediary layers of the epidermis, in chronic renal patients undergoing long-term dialysis treatment, are common and affect the nerve endings, provoking a reduction in the functional capacity, being characterized as a polyneuropathy^(^ 13 ^)^.

In the light of the above, the relationship between income and the above-mentioned adaptation problems is explained, as the low income can be reflected in difficulty in accessing the service, inadequate nutrition, and difficulty in undertaking pharmacological and dialysis treatment^(^ 3 ^)^, which contributes significantly to the inefficacy of the treatment, and consequently in the development of such complications. This fact emphasizes the importance of actions on the part of the professionals so as to meet any needs which these individuals may present. 

The adaptation problem of role failure had a statistical association with age. It is supposed that the relationship of the changes in the role performed by the individual occurs due to the fact that the patients interviewed are of an economically active age, with a mean of 46.6 years old, which strengthens the psychological conflicts in relation to failure in the role which relates to financial life, in which failure to undertake paid activities can even influence the patients' clinical progression. In accordance with the above, authors^(^ 3 ^)^ have identified that the majority of the participants (93.9%) in the economically active age range were not working at the time of the interview. 

The adaptation problem of sexual dysfunction was associated with sex and marital status. In this regard, it stands out that 62.9% of those investigated were in a marital relationship, and that 52.2% were male. Sexual dysfunction in male patients with CKD, in particular, can result from organic and psychological causes. Among the organic causes, neurological, endocrine, hematological, biochemical and pharmacological problems coexist, along with hypertension and diabetes, while psychological causes are related to anxiety, depression, and loss of self-esteem^(^ 4 ^)^. It is known that people at more advanced stages of CKD present the above-mentioned physical and emotional alterations, which trigger sexual dysfunction^(^ 5 ^)^.

The association between marital status and the problem of sexual dysfunction may be related to the fact that, as the majority of the patients are in affective relationships, they are probably sexually active; as a result, they have a higher probability of presenting dysfunction in this area. Furthermore, kidney disease, *per se*, causes reduced libido, which is one of the factors related to this dysfunction^(^ 14 ^)^. In the light of this, social support and counseling regarding the issue are indicated for this clientele, seeking strategies to minimize the problem. 

The impairment of a primary sense: sight was statistically associated with the variable of years of study. One study indicates the association between retinopathy and chronic kidney disease, regardless of the presence of diabetes mellitus, evidencing the link between the microcirculation of the retina and renal compromise^(^ 11 ^)^. As a result, loss of vision in renal patients may be present due to alterations in the ocular microcirculation. However, in relation to years of education, there are no studies indicating a relationship between the impairment of this sense and a low educational level. 

Intolerance to activity also presents a statistical association with the variable of years of education. This adaptation problem, one of the main symptoms of CKD, is evidenced by fatigue and a difficulty in undertaking activities of daily living, and may be related to reduction of red blood cells. Anemia is one of the complications presented by such patients, and is caused by shortage of iron and the relative deficiency of erythropoietin^(^ 15 ^)^. The relationship between intolerance for activity and years of education may be explained by the fact that a low educational level is related to nonadherence to therapy, as the patient does not understand his illness and the importance of the treatment. This fact results in worsening of the complications of CKD, such as anemia, caused by the nonuse of the erythropoietin^(^ 3 ^)^.

Another adaptive problem associated with the variable of years of education was chronic pain, which was also associated with sex. One study which addressed pain in patients with chronic kidney disease indicated that there was no association of the presence of the pain with sex^(^ 16 ^)^. Chronic pain can be related to renal osteodystrophy, one of the complications resulting from renal compromise, manifested by lack of control of the increase of phosphate which provokes bone mineralization. It is a painful condition, and is associated with an increased risk of fracture, with difficulty for self-care, as well as increasing mortality in patients receiving dialysis^(^ 17 ^)^.

A low educational level, a striking characteristic among those investigated in this study, may have repercussions in nonadherence to drug treatment and diet, which can trigger complications in CKD, such as osteodystrophy, a disease which affects the bones and causes intense pains^(^ 14 ^)^. Thus, in the light of the need for appropriate compliance with medications and diet for controlling problems secondary to CKD, such as anemia and renal osteodystrophy, it is fundamental for the multi-professional team to make use of teaching strategies which are reflected in the assimilation of the information received for the control of this clientele's therapeutic regimen. 

The problem of impaired skin integrity was associated with age. The problem of potential for injury was associated with the variables of age and years of education. In consonance with the association mentioned above, one study reveals that the alterations in the skin are related to age progression, as the individual suffers numerous degenerative changes in this organ with advancing age^(^ 18 ^)^. Therefore, it is important for patients and health professionals to have knowledge regarding possible skin alterations in the population with kidney disease, such that these may be recognized at an early stage and treated appropriately. 

Nutrition less than the organism's requirements was associated statistically with age. This problem may be related to the changes in nutritional metabolism, a frequent problem in patients receiving HD^(^ 12 ^)^. A study which evaluated the nutritional aspect of patients receiving hemodialysis, with a mean age of 49.6 years old, revealed a high level of malnutrition in this clientele, reflecting the fact that chronic diseases reduce the desire to ingest food, as well as hindering the absorption of the nutrients^(^ 19 ^)^.

The adaptation problem of hypocalcemia was statistically associated with the variable of access to hemodialysis. Hypocalcemia, the reduction of calcium ions, a complication of CKD, is linked to the fact of poor intestinal absorption of these as a result of the shortage of vitamin D and of the chelatant effect of the phosphorus. In this study, the association of hypocalcemia with the type of access was weak, as the type of access is closely linked with the adequacy of the dialysis treatment. This being the case, definitive access - such as the arteriovenous fistula - is reflected in fewer complications in this clientele^(^ 14 ^)^.

In the light of this fact, the measuring of the calcium must be routine in patients undergoing hemodialysis with any type of vascular access, so as to avoid complications which could even be fatal. 

The impairment in the primary sense: hearing was associated with sex and with the evaluation of urea kinetics. However, a study aiming to characterize the audiological findings in patients with chronic kidney disease did not find significant difference for hearing problems in relation to sex in this clientele^(^ 20 ^)^.

In relation to the kinetic evaluation of the urea (Kt/V), the most important modifiable factor for the survival of patients receiving dialysis is defined as an index of the adequacy of the hemodialysis. When the Kt/V ratio is low, it is reflected in the occurrence of metabolic disorders, which can somehow interfere in the auditory perception of renal patients. Hence one can perceive the need for measuring this index through control systems which quantify, in real-time, how adequate the dialytic process is, so as to prevent or delay possible complications^(^ 14 ^)^.

Finally, the adaptation problem of mobility - gait and/or coordination restricted was associated with the length of time in which the patient had been receiving HD, while the problem of loss of ability in self-care was associated with the duration of the kidney disease and the length of time the person had been receiving hemodialysis.

In this study, the time since diagnosis of CKD and of undertaking hemodialysis therapy had medians of six and four years, respectively. Hence, the long duration of the therapy, as well as of the disease, are reflected in the occurrence of osteomuscular manifestations, which are considered frequent in chronic kidney patients receiving HD. These manifestations are, very often, incapacitating to the extent that they impede or hinder the patient's mobility and his ability to undertake self-care^(^ 14 ^)^. As a result, the rapid action of the nurse in this important problem must be focused upon the prevention and/or minimization of the harm. 

## Conclusion

Based on the statistical associations identified in the variables of the present study, it is concluded that the adaptive problems of the clientele receiving hemodialysis may be influenced by socioeconomic and clinical variables. The variables with the highest rate of association were: Impairment of a primary sense: vision, and years of study; Loss of ability for self-care, and months of hemodialysis; Sexual dysfunction and marital status and sex; Potential for injury, and age; Impairment of a primary sense: touch, and income; Chronic pain and sex; Mobility - gait and/or coordination restricted, and months of hemodialysis. 

There was an association, mainly, in the items of educational level, income, age and sex presented by the clientele. Therefore, one can state that these variables may directly influence the adaptation problems suffered by this population, and that these, as they cannot be changed by nursing, must be discussed and reflected upon with other professionals, with a view to directing the care in accordance with the social context in which the patient is inserted. 

The identification of these associations stands out as a contribution of the study, as the nurse working in nephrology can reflect regarding her care, which is directed to the adaptation problems, as well as towards the socioeconomic and clinical issues, such as to allow the obtaining of positive adaptation in this clientele. 

As a difficulty, this study indicates the shortage of research reporting on the issue studied here, so as to discuss the data for comparison. As a limitation, emphasis is placed on the fact that this study was undertaken only with chronic kidney patients receiving hemodialysis treatment. 
